# Quantitative assessment-based nursing intervention improves bowel function in patients with neurogenic bowel dysfunction after spinal cord injury: Study protocol for a randomized controlled study

**DOI:** 10.1097/MD.0000000000023354

**Published:** 2020-12-18

**Authors:** Qionghua Yin, Can Wang, Jianhong Yu, Qiufang Zhang

**Affiliations:** Department of Intensive Care Unit, The First Affiliated Hospital of Soochow University, Jiangsu, China.

**Keywords:** neurogenic bowel dysfunction, protocol, quantitative assessment-based nursing, spinal cord injury

## Abstract

**Background:**

The neurogenic bowel dysfunction is a kind of familiar sequelae of the spinal cord injury (SCI), occurring in 70 to 80 percent of the SCI patients. The nursing intervention based on quantitative evaluation is to fully consider and assess the disease condition of patients, implement the personalized programs of nursing intervention, meet the patient's nursing needs to the maximum extent, improve the quality of nursing, and then facilitate the rehabilitation of patients. Our aim is to implement this program to evaluate the impact of this nursing intervention based on quantitative evaluation on the quality of life and bowel function in the neurogenic bowel dysfunction patients after SCI.

**Methods:**

The experiment is a randomized clinical research which will be implemented from May 2021 to October 2021 at the First Affiliated Hospital of Soochow University. The experiment was granted through the Research Ethics Committee of the First Affiliated Hospital of Soochow University (No.100238765). Fifty neurogenic bowel dysfunction patients after SCI confirmed via the imaging are included in this study. The patients with the history of bowel diseases or patients who are unwilling to cooperate with the evaluation will be excluded. The primary outcomes are bowel function recovery and satisfaction of the patients. The secondary outcomes are quality of life evaluated by SF-36 questionnaire. The questionnaire involves physical pain, role physiology, physiological functions, social functions, vitality, general health, mental health and role-motional.

**Results:**

Comparison of clinical parameters between the 2 groups will be shown in Table 1.

**Conclusion:**

Nursing intervention based on the quantitative evaluation can improve the quality of life and recovery of intestinal function for the neurogenic intestinal dysfunction patients after SCI.

**Trial registration number:**

researchregistry6143

## 1 Introduction

The neurogenic bowel dysfunction is a kind of familiar sequelae of the spinal cord injury (SCI), occurring in 70% to 80% of the SCI patients.^[1,2]^ For patients with SCI, this is a significant problem, which will cause adverse effects on mental, social and physical health.^[3–5]^ When patients with SCI are asked to list the adverse effects on their quality of life in order of importance, intestinal dysfunction ranked second. Intestinal dysfunction secondary to SCI can cause urinary incontinence, uncoordinated defecation and constipation.^[6,7]^ The degree of dysfunction may be influenced via the external factors or the internal features of SCI itself, and may cause social or personal economic burden.^[8,9]^ Therefore, the attention should be focused on identifying the effective interventions to improve the intestinal function.

Generally, the treatment of intestinal function has attracted more and more attention, despite the relevant research is still very few in this field. In recent years, nursing intervention has increasing influence on the quality of life and intestinal function in the neurogenic bowel dysfunction patients.^[10]^ The traditional nursing approaches are conformist, focusing only on the basic nursing and treatment of the diseases, ignoring the effect of this disease on the quality of treatment and nursing. Hence, traditional nursing has been unable to meet the psychological and physiological needs of patients. The former investigations have indicated that the nursing intervention based on quantitative evaluation is to fully consider and assess the disease condition of patients, implement the personalized programs of nursing intervention, meet the patient's nursing needs to the maximum extent, improve the quality of nursing, and then facilitate the rehabilitation of patients.^[11]^ However, no reliable evidence has been reached due to the poor study design. Our aim is to implement this program to evaluate the impact of this nursing intervention based on quantitative evaluation on the quality of life and bowel function in the neurogenic bowel dysfunction patients after SCI.

## 2 Materials and methods

The experiment is a randomized clinical research which will be implemented from May 2021 to October 2021 at the First Affiliated Hospital of Soochow University, in accordance with the purposes of the Declaration of Helsinki. The experiment was granted through the Research Ethics Committee of the First Affiliated Hospital of Soochow University (No.100238765) and recorded in research registry (researchregistry 6143). The recruited patients are given the written informed consent before registration. Fifty neurogenic bowel dysfunction patients after SCI confirmed via the imaging are included in this study. The patients with the history of bowel diseases or patients who are unwilling to cooperate with the evaluation will be excluded. Via utilizing the number table, all the patients involved in the experiment are assigned a random number in a random envelope, and the allocation results are hidden from them. The patients are randomly divided into control group (25 cases) and study group (25 cases).

### 2.1 Nursing in both groups

In control group, the patients are given the routine nursing, containing psychological nursing and the education of disease health. The patients are guided to eat more foods with high dietary fiber, fresh fruits and vegetables, drink more water, facilitate intestinal patency, and eat less candies and milk that are prone to gas production, so as to decrease abdominal distension.

Nursing in study group involves:

(1) Quantitative evaluation: on the basis of the imaging examination results and clinical symptoms, the patients are scored in severe, moderate and mild.
(2) Individualized nursing intervention:
  a. Reduce the intake of high-protein and high-fat food. If conditions permit, the daily intake of liquid should be at least 2000 ml.
  b. Implement an abdominal massage to speed up defecation. While massaging, patients are in the supine position. Centering on the navel, the nurse utilizes one or both hands’ fingers to circle the anatomical position of the colon from right to left, lasting for twenty minutes.
  c. Exercise muscles involved in the defecation: guide the patients to exercise their abdominal muscles. The patient is in the bed slope position or sitting on the toilet. Abdominal muscles are trained via abdominal breathing, deep breathing as well as defecation. Instruct the patient to perform the anal contraction exercises correctly. The patients’ knees slightly flexed apart with legs together and is in a supine position. Subsequently, patients raise buttocks and then contracts anus as instructed. In the process of anus contraction, the thigh muscle and perineal muscle contract for 6 seconds and relax for 4 seconds.
  d. Stimulate anus, facilitate colorectal reflection: the action of anal can stimulate intestinal peristalsis. The nurse put on gloves, lubricate middle finger and index finger with the liquid paraffin, inserts into anus slowly and then rotates in rectum. Anal action is implemented daily for 2 minutes.

### 2.2 Outcome

The primary outcomes are bowel function recovery and satisfaction of the patients. The secondary outcomes are quality of life evaluated by SF-36 questionnaire.^[12]^ The questionnaire involves physical pain, role physiology, physiological functions, social functions, vitality, general health, mental health, and role-motional.

### 2.3 Statistical analysis

The analysis of all data are carried out with the IBM SPSS Statistical software Windows version 20 (IBM Corp., Armonk, NY). Afterwards, all the data are described with appropriate characteristics such as mean, median, standard deviation as well as percentage. Continuous and categorical variables are analyzed using the independent *t*-tests and *χ*2-tests. For the significance level, the value of *P* needed to be less than .05.

## 3 Result

Comparison of clinical parameters between the 2 groups will be shown in Table 1.

**Table 1:** Comparison of clinical parameters between the 2 groups.

Study group (n = 25)	Control group (n = 25)	*P* value
Bowel function		
Abdominal distension		
Constipation		
Defecation time		
Fecal incontinence		
Quality of life		
Physical functioning		
Role-physical		
Bodily pain		
General health		
Vitality		
Social functioning		
Role-motional		
Mental health		

## 4 Discussion

The bowel dysfunction is one of the most familiar problems in the patients with SCI.^[13,14]^ In the early injury stage, the anal sphincter and rectum internal layer are in the stage of total incontinence. After the acute phase, the voiding reflex recovers with the recovery of the lumbosacral central spinal cord.^[15]^ The right side of colon and the bowel are peristalsis supplied by the vagal innervation, so that the feces can be transported to large intestine. After the SCI, patients may have several problems, for instance, reduced sensation and muscle strength, and impaired respiratory as well as other functions.^[16]^ Specifically, SCI can lead to the high spasticity and paralysis of anal sphincter, or lead to the voiding dysfunction. The participation of pelvic and abdominal muscles and effective movement of bowel are essential for the normal defecation. According to the severity of intestinal dysfunction and SCI, we develop a set of nursing intervention based on the quantitative evaluation for the intestinal dysfunction patients after SCI, so as to make nursing more efficient and targeted. Nevertheless, insufficient research design and small size of sample are the limitations of this experiment. The effectiveness of nursing intervention based on quantitative evaluation needs further study.

## 5 Conclusion

Nursing intervention based on the quantitative evaluation can improve the quality of life and recovery of intestinal function for the neurogenic intestinal dysfunction patients after SCI.

## Author contributions

Qiufang Zhang designs the protocol. Can Wang reviews the protocol. Jianhong Yu performs data analysis. Qionghua Yin finishes the manuscript. All authors approve the submission.

**Data curation:** Can Wang.

**Formal analysis:** Can Wang.

**Funding acquisition:** Qiufang Zhang.

**Resources:** Jianhong Yu.

**Software:** Jianhong Yu.

**Writing – original draft:** Qionghua Yin.
